# Nodes staging score to quantify lymph nodes for examination in gastric cancer

**DOI:** 10.1097/MD.0000000000021085

**Published:** 2020-08-14

**Authors:** Liping Sun, Qiaohong Liu, He Ren, Ping Li, Gang Liu, Lining Sun

**Affiliations:** a Shanghai University of Medicine & Health Sciences, Shanghai; b School of Mechanical and Electrical Engineering, Soochow University, Suzhou, P.R. China.

**Keywords:** gastric cancer, lymph node invasion, model, NSS

## Abstract

The lymph nodal invasion diagnosis is critical for therapeutic-decision and follows up in gastric cancer. However, the number of nodes to be examined for nodal invasion diagnosis is still under controversy, and the model for quantifying risk of missing positive node is currently not reported yet. We analyzed the nodal invasion status of 13,857 gastric cancer samples with records of primary tumor stage, the number of examined and positive lymph nodes in the surveillance, epidemiology, and end results (SEER) database, fitting a beta-binomial model. The nodes need to be examined with different primary tumor stage were determined based on the model. Overall, examining 11 lymph nodes reduces the probability of missing positive nodes to <10%, and the currently median nodes dissected is adequate (12 nodes). While the number of nodes demands to be dissected for T1, T2, T3, and T4 subgroups are 6, 19, 40, and 66, respectively. The currently implemented median value for these samples was 12, 12, 13, and 16, separately. It implies that the number of nodes to be examined is sufficient for early gastric cancer (T1), but it is inadequate for middle and advanced gastric cancer (T2–T3). The clinical significance of nodal staging score was validated with survival information. In summary, we first quantified the lymph nodes to be examined during surgery using a beta-binomial model, and validated with survival information.

## 1 Introduction

Gastric cancer is among most prevalent cancers worldwide, with 679,100 new cases and subsequently 498,000 deaths due to gastric cancer in China, 2015.^[1]^ Radiation is currently one of the most important adjuvant therapy methods. Patients with lymph nodal invasion significantly benefit^[2–4]^ from radiation, while not for lymph node-negative patients. On the other hand, drawbacks of radiation have been widely reported, such as secondary malignancy.^[5,6]^

As lymph nodal invasion status is one of the important clinical indicators for post-operational radiation therapy, adequate lymph nodes dissection and examination are necessary for accurate diagnosis. According to previous studies, pre-operative staging is an important indicator for guiding lymph nodal dissection.^[7]^ Report suggests that at least 18 lymph nodal should be warranted for advanced node-negative gastric cancer,^[8]^ while another two studies suggest 15.^[9,10]^ However, the exact number of nodal to be examined for patients with different primary tumor stages has not been reported hitherto.

In our work, the relationships between missing positive possibility and tumor stage was investigated using the number of examined lymph nodes and invaded nodes from the surveillance, epidemiology, and end results (SEER) database (N = 13,857), fitting a beta-binomial model. Our results indicate that the minimum number of nodes examined for primary tumor (T1–T4) were 6, 19, 40, and 66, respectively, to reach <10% probability of missing nodal.

## 2 Results

### 2.1 Data profile

The data is all retrieved from SEER database, thus no ethics committee or institutional review board approval is needed for this study. After pre-processing of records in the SEER database, 13,857 samples were involved in this study. The detailed information of primary tumor locations and histological type was shown in Table 1, Supplemental Digital Content (Appendix Table 1). The sample number increases along with the primary tumor stage. The number of average examined nodes in T1 to T4 ranged from 14.3 to 19, and the median of examined nodes was 12 to 16.

**Table 1:**
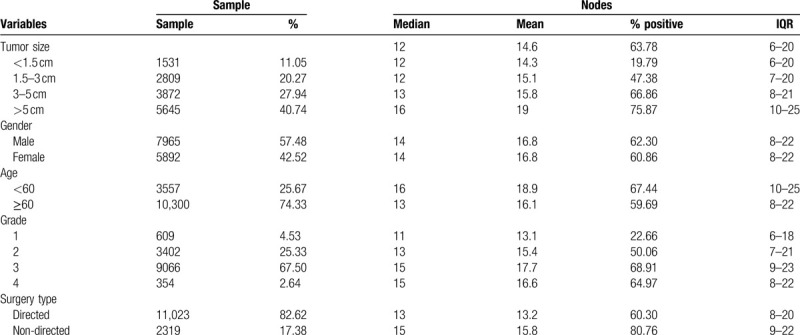
General information of samples involved.

### 2.2 False negative diagnoses in overall data

**Figure 1:**
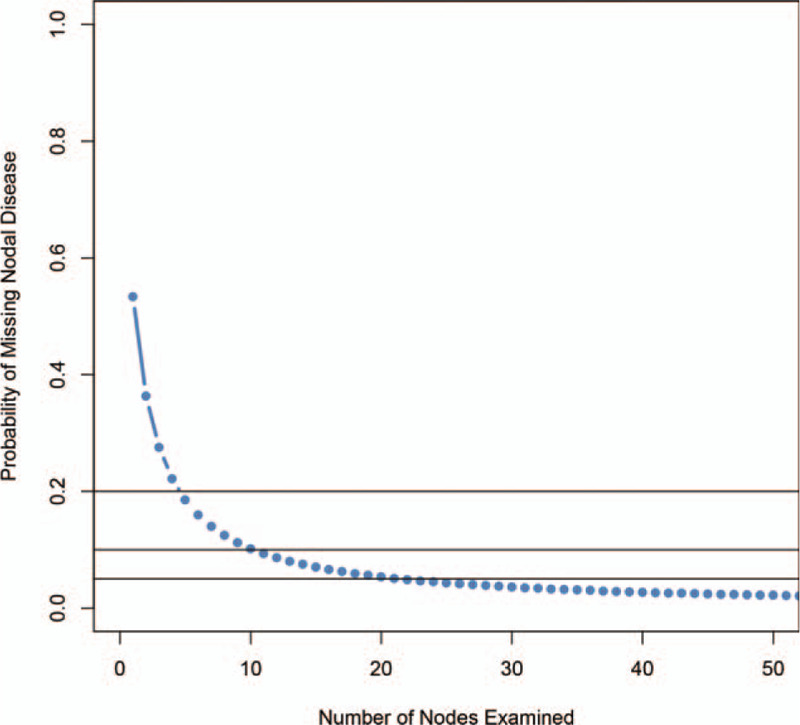
Sensitivity of nodal diagnosis according to the nodes examined. The *x*- and *y*-axis indicates the number of node examined and probability of missing positive nodes.

Using our model, the parameters of beta-binomial model, α and β, were estimated to be 1.0058 (95% CI 0.9724–1.0402) and 1.1518 (95% CI 1.1100–1.1950). The overall probability of missing nodal invasion (1-sensitivity), as a function of the counts of examined nodes was calculated, as shown in Figure 1. The probability of missing nodal was decreasing along with the increasing of examined nodes, as expected. When one lymph node was examined, the probability of missing nodal was 52.38%. The minimum number of nodes that need to be examined was 5 for reaching <20% missing nodes possibility, and 11/22 for 10%/5%, respectively (Detailed information in Supplemental Digital Content [Appendix Table 2]). The median value of current examined nodes was 12, and the corresponding probability of missing nodal was 8.65%, suggesting that the current nodes examined are adequate for overall samples.

### 2.3 False negative diagnoses in different primary stage

**Figure 2:**
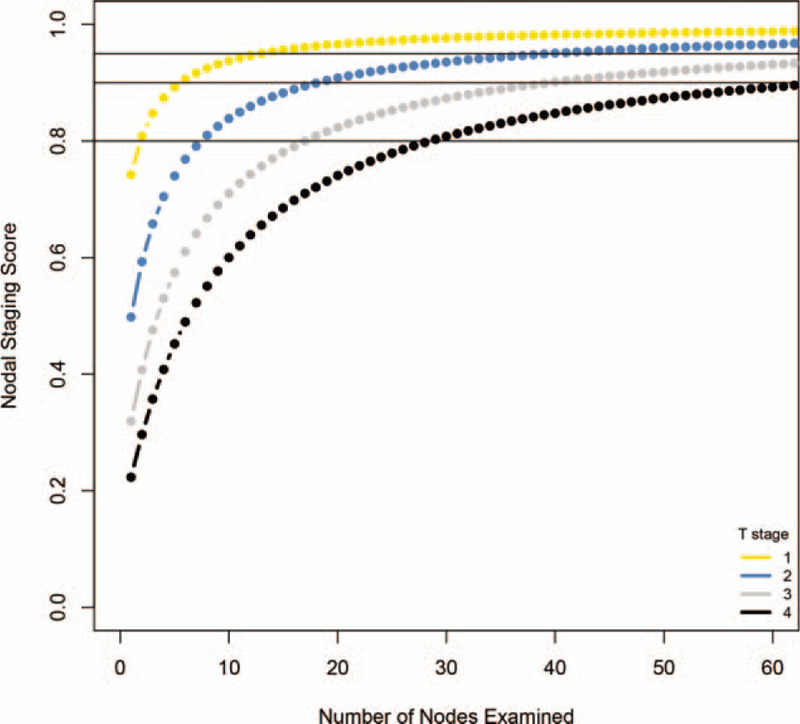
Sensitivity of nodal diagnosis (*y*-axis) in different primary tumor stage according to the nodes examined (*x*-axis).

The primary tumor stage is the most important indicator for nodes examination. The probability of missing nodal was also estimated in different primary tumor stage (T1–T4). To minimize the probability of missing nodal below 20%, the least number of examined nodes are 2, 8, 17, and 29 for T1, T2, T3, and T4, respectively (Fig. 2). While lowering the probability to 10%, the nodes numbers are 6, 19, 40, and 66 for T1 to T4. When the examined nodes number is 12 (the current median value of examined nodes), the probabilities of missing nodes are 5.32%, 14.03%, 25.67%, and 36.09% for T1 to T4. The median of examined nodes numbers for T1 to T4 are 12, 12, 13, and 16 (Table 1) and the corresponding probabilities of missing nodal are 5.32%, 14.03%, 24.29%, and 30.19%, respectively.

Combining current node positive rates of primary tumor stages and that of our results, the corrected rates were calculated (Table 2). The corrected node-positive rates are 19.60%, 17.96%, 13.11%, and 10.10% higher than observed rates for T1 to T4, respectively.

**Table 2:**
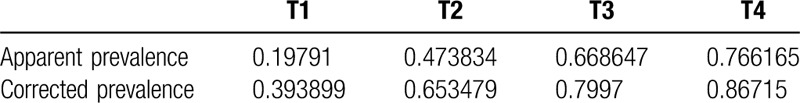
Observed and corrected prevalence of node-positive rates.

To test the robustness of the results, we retrieved 80% samples from the original data and assessed the number of nodes need to examine, and repeat for 1000 times. The results showed that the median nodes to be examine to reach <10% missing nodes possibility for different primary tumor stage was 6 (95% CI: 5.2–7.1), 19 (95% CI: 17.1–20.0), 39 (95% CI: 34.5–43.2) and 67 (95% CI: 65.3–73.5), which consistent with our results.

### 2.4 Nodal staging score and survival

**Figure 3:**
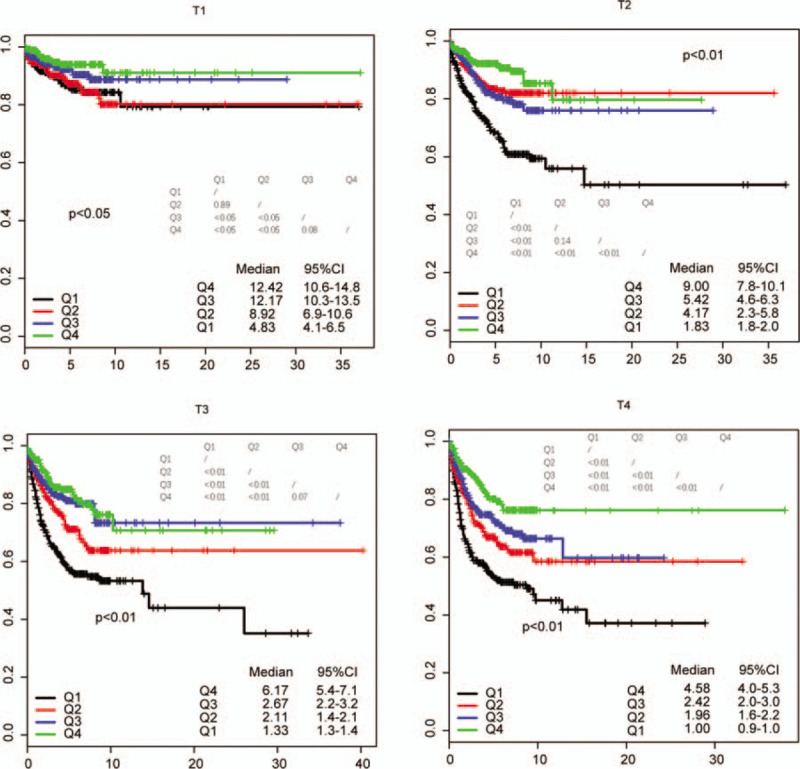
The survival of patients in different primary tumor stage according to the nodal staging score. Q1 to Q4 indicate the lowest-highest nodal staging score.

The follow-up information was not used in our model, and it is independent from our nodal staging score (NSS). The NSS in diagnosed N0 stage (no invaded lymph nodes detected) in primary tumor stages was divided by quantiles, and survival difference was compared (Fig. 3). The NSS is significantly associated with survival in all these four categories (T1–T4).

### 2.5 Other clinical information and NSS

In addition to primary tumor stage, differentiation grade was also used to evaluate the NSS. To reach <10% nodal missing possibility, 7, 19, 39, and 36 nodes need to be examined (Supplemental Digital Content [Appendix Table 3]). The result indicates that the differentiation is also an important indicator for nodal dissection.

## 3 Discussion

Lymph node invasion is strongly associated with recurrence, metastasis, and poor prognosis in gastric cancer.^[11,12]^ Hence, lymph node invasion is crucial for therapeutic decision-making of gastric cancer patients, including radiation therapy selection and drug usage.^[2,13]^ However, the number of examined lymph nodes is controversial, as inadequate nodes examination may lead to the miss of invaded nodes, while excessive number of nodes examination would reduce the immune activity. In the past years, efforts have been devoted to optimize the examined nodes.^[14–17]^ However, the exact nodes number required for dissection in gastric cancer is not suggest. Using beta-binominal model, our work has shown that currently implemented number of nodes dissected is sufficient for early gastric cancer (T1) but not for middle and advanced gastric cancer (T4).

In this study, we chose primary tumor stage as the indicator to determine the number of lymph nodes to be dissected and examined. In present model, a few hypotheses were employed. First, the beta-binomial distribution of nodal invasion is assumed. However, the lymph nodes invasion rate of disease on different anatomic locations varies, which may have brought bias for the model. Experimentally, we did not see the lack of the model, based on previous studies in other cancers.^[18–21]^ Another assumption in this work is that all examined nodal status is correct, which means, no false positive or false negative exist. This is considered to be reasonable as it is claimed by SEER database that all diagnoses were made by expertise pathologists. Clinical information including pathological information impact on cancer development and migration predict nodal invasion. Results of NSS based on differentiation grade were consistent with this.

Limits of this study also exist. It is also a retrospective study, though multicenter. Important clinical variables, such as drug usage, time to metastasis/recurrence, and surgery type are not available for this study, which makes the lack of enrollment control and may bring some bias. On the other aspect, the impact the NSS on treatment, including adjuvant therapy/neoadjuvant therapy cannot be found in SEERstat database, which makes the implication evaluation of NSS on therapy unavailable.

## 4 Methods and Materials

### 4.1 Data collection

The SEER database includes 17 registries covering 26% population in the United States. Only samples with gastric cancer as the primary cancer were retained (resident tumor). Patients without records related to number of regional nodes examination, or positive nodes number were discarded. Furthermore, records with missing values in all these entities were also excluded. The primary tumor stages were divided into four categories (termed T1, T2, T3, and T4, separately). Totally, 13,857 samples enrolled in this study.

Limitation of this study is that the data retrieved from SEER database was retrospective and detailed clinical variables, including time to metastasis and local recurrence, were not included in the records. Thus, recurrence-free survival and metastasis-free survival information are unavailable with this dataset.

### 4.2 Statistical analysis

A previously described mathematical model was adopted^[20]^ using the beta-binomial distribution to estimate the possibility of missing the invasion-positive as a function of total lymph nodal dissected and examined. The true positive indicates that the true status of lymph node was invaded, and the results showed at least one lymph node was invaded. The true negative means that the true status was uninvaded, and the examination results also showed no lymph invasion. The false negative defines that the true status is invaded, while the results showed no invasion.

Three hypotheses employed in this model:

1. All nodes examined are correct, which means, no false-negative or false-positive result exist.
2. The distribution of lymph nodes is exchangeable. That means, the possibility of the examined lymph nodes has all the same chance to be invaded, which enables us to calculate the invasion possibility across patients. Biologically, this assumption is not reasonable, especially in small studies with different clinicopathological characteristics. However, if a large cohort employed, the assumption is reasonable, as most previous studies stated.
3. The sensitivity of true positive and false negative is same, which enables us to generalize the results to pathologically node-negative samples. Sensitivities only can be calculated in node-positive samples.

The procedure of model development and validation:

1. The proportion of the number of positive lymph nodes (non-N0 stage) and the number of total nodes dissected/examined was used to estimate the coefficients of beta-binomial (α and β) distribution. In this step, samples were limited to these with at least two lymph nodes examined, because by definition, the node-positive rate would be 100% if only one lymph node examined. In gastric cancer, primary tumor stages are used as indicators for surgery and adjuvant therapy.^[22–24]^
2. False-negative rates were estimated according to the model and coefficient estimated, in the overall datasets and sub-datasets (different primary tumor stage), and the observed and corrected prevalence was calculated using the following function: 
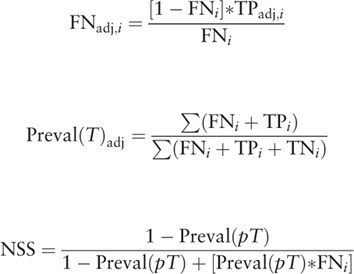
  where FN_adj,*k*_ indicates adjusted false negatives; FN is observed false negative; TP_adj,*k*_ is the number of true positive; *T* indicates the primary tumor stage categories. FN, TP, TN means false negative, true positive, true negative, respectively.
3. Considering overall survival information is independent from lymph nodes dissection and NSS, we used it for validation. Tumors with no lymph nodes invasion was selected. Samples of each primary tumor stage category (T1–T4) were divided based on the quartile of NSS, and survival difference was compared using Log Rank test.

### 4.3 Software

All analyses involved in this study were implemented on R software (v3.1.0) and R packages. The VGAM (v1.0-3) and bbmle (v1.0.18) package were used to evaluate α and β parameters of beta-binomial model. The survival analysis was completed with R package “survival” using Kaplan-Meier method.

## Author contributions

Liping Sun, Qiaohong Liu and He Ren paticipate in experimet design, Liping Sun and Lining Sun is responsable for funding acquisition, Liping Sun, Qiaohong Liu, He Ren and Ping Li analyzed and visualized the data, all authors intepreted the results, wrote the draft and revised the manuscript.

## Supplementary Material

Supplemental Digital Content

## Supplementary Material

Supplemental Digital Content

## Supplementary Material

Supplemental Digital Content
